# Multi-Country Analysis of Treatment Costs for HIV/AIDS (MATCH): Facility-Level ART Unit Cost Analysis in Ethiopia, Malawi, Rwanda, South Africa and Zambia

**DOI:** 10.1371/journal.pone.0108304

**Published:** 2014-11-12

**Authors:** Elya Tagar, Maaya Sundaram, Kate Condliffe, Blackson Matatiyo, Frank Chimbwandira, Ben Chilima, Robert Mwanamanga, Crispin Moyo, Bona Mukosha Chitah, Jean Pierre Nyemazi, Yibeltal Assefa, Yogan Pillay, Sam Mayer, Lauren Shear, Mary Dain, Raphael Hurley, Ritu Kumar, Thomas McCarthy, Parul Batra, Dan Gwinnell, Samantha Diamond, Mead Over

**Affiliations:** 1 HIV, TB and Health Financing, Clinton Health Access Initiative, Boston, Massachusetts, United States of America; 2 Planning, Monitoring, Evaluation and Research, National AIDS Commission, Lilongwe, Malawi; 3 Department for HIV and AIDS, Ministry of Health, Lilongwe, Malawi; 4 Community Health Sciences Unit, Ministry of Health, Lilongwe, Malawi; 5 Department of Planning and Policy Development, Ministry of Health, Lilongwe, Malawi; 6 National ART Program, Ministry of Health, Lusaka, Zambia; 7 Department of Economics, University of Zambia, Lusaka, Zambia; 8 Planning, Monitoring and Evaluation Division, Ministry of Health, Kigali, Rwanda; 9 Planning, Monitoring and Evaluation Directorate, Federal HIV/AIDS Prevention and Control Office, Addis Ababa, Ethiopia; 10 HIV/AIDS, TB and MCWH, National Department of Health, Pretoria, South Africa; 11 HIV, TB and Health Financing, Clinton Health Access Initiative, Lilongwe, Malawi; 12 HIV, TB and Health Financing, Clinton Health Access Initiative, Kigali, Rwanda; 13 HIV, TB and Health Financing, Clinton Health Access Initiative, Addis Ababa, Ethiopia; 14 HIV, TB and Health Financing, Clinton Health Access Initiative, Lusaka, Zambia; 15 HIV, TB and Health Financing, Clinton Health Access Initiative, Pretoria, South Africa; 16 Center for Global Development, Washington, District of Columbia, United States of America; University of British Columbia, Canada

## Abstract

**Background:**

Today's uncertain HIV funding landscape threatens to slow progress towards treatment goals. Understanding the costs of antiretroviral therapy (ART) will be essential for governments to make informed policy decisions about the pace of scale-up under the 2013 WHO HIV Treatment Guidelines, which increase the number of people eligible for treatment from 17.6 million to 28.6 million. The study presented here is one of the largest of its kind and the first to describe the facility-level cost of ART in a random sample of facilities in Ethiopia, Malawi, Rwanda, South Africa and Zambia.

**Methods & Findings:**

In 2010–2011, comprehensive data on one year of facility-level ART costs and patient outcomes were collected from 161 facilities, selected using stratified random sampling. Overall, facility-level ART costs were significantly lower than expected in four of the five countries, with a simple average of $208 per patient-year (ppy) across Ethiopia, Malawi, Rwanda and Zambia. Costs were higher in South Africa, at $682 ppy. This included medications, laboratory services, direct and indirect personnel, patient support, equipment and administrative services. Facilities demonstrated the ability to retain patients alive and on treatment at these costs, although outcomes for established patients (2–8% annual loss to follow-up or death) were better than outcomes for new patients in their first year of ART (77–95% alive and on treatment).

**Conclusions:**

This study illustrated that the facility-level costs of ART are lower than previously understood in these five countries. While limitations must be considered, and costs will vary across countries, this suggests that expanded treatment coverage may be affordable. Further research is needed to understand investment costs of treatment scale-up, non-facility costs and opportunities for more efficient resource allocation.

## Introduction

The availability of HIV care and treatment services worldwide has expanded rapidly. This is reflected in the massive scale-up of people on antiretroviral therapy (ART) from 400,000 people on treatment in 2003 to nearly 13 million at the end of 2013 [1], [2]. This success was accompanied by an unprecedented increase in resources, with annual funding levels increasing from $5 billion in 2003 to $19.1 billion in 2013. Despite this achievement, under the 2013 WHO HIV Treatment Guidelines, less than half of eligible people living with HIV are currently receiving treatment (*ibid*).

While governments are committed to achieving universal access to treatment for those in need, funding for HIV has begun to flat-line, with international contributions declining for the first time in 2010 and increasing more gradually in the years since [3]. Additional funds may be needed for the HIV response, but an equally important priority is making high-quality treatment as affordable as possible and optimizing the allocation of existing HIV funding across interventions. This will maximize the impact of each dollar spent, reducing the size of the resource gap and encouraging increased investments from donors and national governments.

Planning for and meeting these ambitious commitments requires a better understanding of the current costs of treatment and the identification of potential areas where efficiency gains are possible. This study aimed to fill an important evidence gap around these critical questions. While previous studies have examined treatment costs within particular country contexts, or at sites funded by specific funding streams, this is one of the first multi-country studies to examine a random sample of ART sites across a wide range of funding streams, facility sizes, and locations.

## Methods

### Setting

Data collection was conducted between 2010 and 2011 in 161 facilities across five countries - Ethiopia, Malawi, Rwanda, South Africa, and Zambia. The study included a retrospective assessment of facility-level HIV care and treatment unit costs across facilities, conducted from the payer perspective, or that of governments and donors. There was also an accompanying patient outcome analysis. Data was collected for a 12-month period, or ‘cost data year’, which was determined for each country based on the most recently available full year of treatment data. Cost data years ranged between 2009 and 2011 depending on the country.

Countries were selected to improve understanding of variations in costs across high-burden contexts that were in different financing situations and at different stages of the HIV response. In 2009, at the time of country selection, Rwanda was receiving significant donor funding, while Malawi faced funding threats following the rejection of the Round 10 Global Fund submission. Rwanda was also nearing universal access to treatment, while in South Africa, less than 60% of people eligible for treatment were enrolled in ART [4]. It is important to note that when the study began in these countries eligibility for treatment was shifting from those with a CD4 count less than 200 cells/mm^3^, per the 2006 WHO HIV Treatment Guidelines, to those with a CD4 count less than 350 cells/mm^3^, per the 2010 WHO HIV Treatment Guidelines.

Facilities were selected using stratified random sampling. Two to three characteristics for stratification were prioritized for each country depending on their local relevance. This included facility size or type (small clinic vs. large hospital), location (rural vs. urban), and/or funding stream (US Government President's Emergency Plan for AIDS Relief vs. Global Fund to Fight AIDS, TB and Malaria vs. Ministry of Health). The representation per stratum was determined by first examining the proportion of patients and then the proportion of total sites included. Each site was assigned a random number using the RAND function in Microsoft Excel 2007 and sites were ordered and selected accordingly. Where necessary, clustered sampling was used, sampling first for “parent” facilities that provided certain services to a group of smaller centers (e.g., laboratory services through sample transport), and then sampling for these “child” facilities. Thirty facilities were selected in each country, with the exception of Ethiopia where a larger sample (41 facilities) was recommended by the Government and local partners to better represent the context. Table 1 includes descriptive information on the facilities included in the study sample.

**Table 1:** Sample Characteristics.

	Indicator	Rwanda	Malawi	Ethiopia	Zambia	South Africa (RSA)
**N (Nb of Sites)**	Sampled Sites	30	30	41	30	30
	Total Sites	286	222	397	385	1,095
**Patients on ART**	ART Patients at Sampled Sites	22,886	48,276	37,335	32,004	47,370
	Total ART Patients	74,357	226,462	190,606	367,498	1,132,913
**Type**	Health Center or Clinic	23	18	21	18	19
	Hospitals	7	12	20	12	11
**Location**	Urban/Peri-Urban	6	11	31	16	18
	Rural	24	19	10	14	12
**Years of ART**	Median Years of ART	8.3	7.7	7.8	8.3	7.3
**Service Provision**	(Min–Max)	(4–11)	(4–10)	(5–11)	(5–14)	(3–11)
**Integration of Services**	Dedicated (HIV-Only)	1	3	0	10	2

### Cost data collection

For the purpose of this study, care and treatment were defined as the full range of facility-level, HIV-related medical services provided to the patient from the time the patient is enrolled on ART. Specifically, this included the costs of antiretroviral medications (ARVs), opportunistic infection medications (OIs), laboratory costs, nutritional support, direct and indirect personnel, facility-level training, equipment, clinical and non-clinical supplies, building maintenance and other administrative support costs. Equipment, building and supply costs were amortized. The costs of adherence and other support programs were captured where they were incurred at the facility (e.g., community health workers were paid through the facility). Treatment-related costs incurred outside of primary facilities, such as lab costs at tertiary facilities associated with diagnosis of opportunistic infections or patient monitoring, were also included.

However, HIV testing before ART initiation and treatment-related costs outside of the facility were not captured. For example, medical care (e.g., inpatient care) delivered at referral sites would have required patient-level or cohort analysis. Laboratory sample transport and supply chain costs, which were not accounted for in commodity pricing, were excluded. The study did not capture the cost of supervision or other associated managerial and support for facilities implemented above the facility-level by the government, donors or implementing partners. Non-medical interventions such as outreach, income-generating activities and interventions for orphans and vulnerable children were also excluded.

Data sources included retrospective program records such as patient registers, account ledgers, pharmacy stock cards and other facility documentation. Once cost data was collected for the facility, the unit cost per patient-year was derived through the allocation described below:

1. *Allocation to HIV treatment*: Since few facilities in the sample specialized in HIV-related treatment services, input costs shared with other services were allocated to HIV treatment based on the proportion of HIV visits relative to all outpatient visits and other relevant measures (e.g., proportion of building area dedicated to HIV).
2. *Allocation to patient type*: Allocation of costs to adult ART patients, as opposed to pre-ART and pediatric patients (patients under 15 years old), was performed using the proportion of patients in each category, adjusted by visit intensity where appropriate. The allocation was based on patient records (e.g., registers, pharmacy records and electronic medical records), and supplemented by interviews with staff where necessary.
3. *Derivation of cost per patient-year*: The total allocated HIV costs for ART patients was then divided by the total number of ART patient-years over the same one-year timeframe to measure the cost per ART patient-year at a given facility.

This “top-down” allocation was replaced with a “bottom-up” or normative calculation of ARV costs where there were severe data limitations. ARV costs were generally measured by calculating facility consumption from initial and final stock on pharmacy stock cards, and then allocating total consumption to different patient types as described above. However, where stock card data was considered unreliable or of poor quality, the final ARV cost ppy was calculated using the site's regimen mix and a normative cost per patient-year by regimen. Therefore the facility-level expenditure after stock-outs and product expiry was not captured consistently.

Local currency was converted to US dollars using the average conversion rate during the survey period. Nominal costs for each cost data year are presented here.

### Patient outcomes data collection

Patient outcomes data was collected by randomly selecting charts for 50 new adult patients and 50 established adult patients at almost all facilities. Smaller or newer sites did not have 50 eligible patient files and therefore the maximum available number was collected. In addition, one site in Rwanda was excluded from the outcome analysis, as there were no eligible charts available.

Charts were chosen by selecting every “nth” chart from the ART register or the physical files where no register was available. Where patient data was collected in an electronic medical records system, a random sample was extracted from the electronic data instead of a paper register or charts. New patients were defined as patients who had initiated ART within the eighteen month window of one year prior to the beginning of the cost data year to six months into the cost data year. Established patients were defined as those who had initiated ART at any point prior to one year before the cost data year. Charts were excluded if the patient was less than 15 years old. In addition, charts were excluded if the patient was classified as lost to follow-up or had not visited the facility in three months, stopped treatment or transferred between facilities during the cost data year. Charts were only excluded for missing information if the record was missing the date of ART initiation or initiation criteria.

A set of basic patient indicators was extracted from the charts, including characteristics such as age and sex; details regarding patient initiation into ART (date of initiation, starting regimen, CD4 count and/or viral load where available); and the last known status and date of last visit to the facility.

Retention was measured as the percentage of patients that were not transferred to another facility and were maintained alive and on treatment after 12 months. For new patients, this was measured as a retention rate from the ART initiation date. For established patients, this was measured as an attrition rate from the start of the cost data year. This meant that any established patient alive and on ART at the end of the cost data year would have received at least two full years of treatment.

### Data analysis

Initial sampling and facility-level cost and outcome calculations were performed using Microsoft Excel 2007 and all analysis was then conducted using STATA IC13. As discussed above, a “top-down” allocation was conducted to arrive at one unit cost per patient-year by facility. The simple mean was then calculated across all facilities in the sample for each country. A weighted mean was calculated by weighting facility unit-costs by the number of ART patient-years per facility in the cost data year. To determine significance, authors used the Fisher's F Test. Statistical significance was set at p<0.05.

### Ethical considerations

Ethical approvals for the project were secured from the appropriate national research review boards and in some cases, sub-national bodies, in the five study countries. This included the Rwanda National Ethics Committee, Malawi National Health Sciences Research Committee, South Africa Human Sciences Research Council, Ethiopia Health and Nutrition Research Institute Scientific and Ethical Review Committee and University of Zambia Biomedical Research Ethics Committee. All patient data were anonymized prior to analysis.

## Results

### ART program unit cost summary

**Figure 1:**
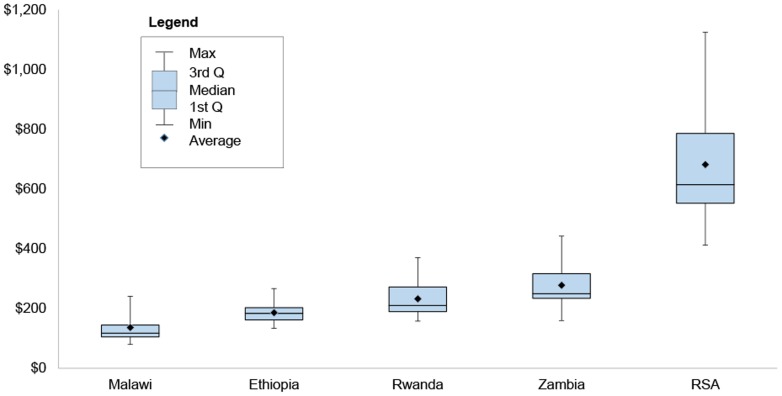
Cost per ART Patient-Year by Country (USD).

**Figure 2:**
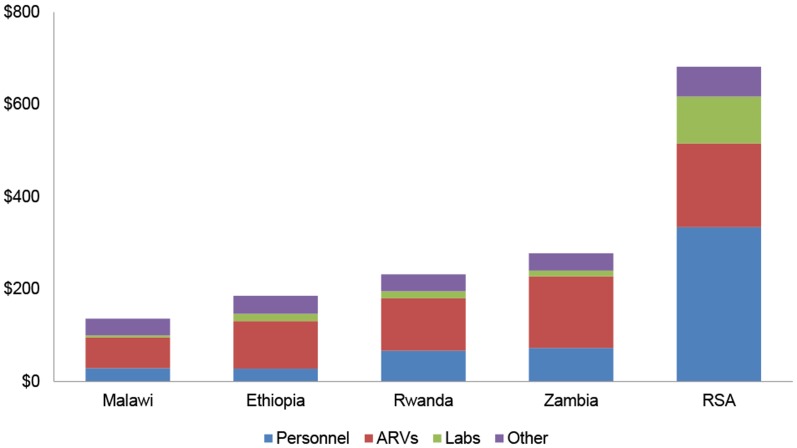
Simple Average Cost per ART Patient-Year by Country by Cost Category (USD).

The simple mean cost per patient-year across the four lower and lower middle income countries (Ethiopia, Malawi, Rwanda and Zambia) is $208 ppy, with higher costs seen in South Africa ($682 ppy). The facility-level costs of delivering ART per patient-year (ppy) were found to be $136 in Malawi (95% CI $119–$154); $186 in Ethiopia (95% CI $175–$196); $232 in Rwanda (95% CI: $208-$257); $278 in Zambia (95% CI: $251-$305); and $682 in South Africa (95% CI $615–$749). Table 2 and Figures 1–2 present a summary of these costs.

**Table 2:** Cost of Treatment per Patient-Year by Country (USD).

		ARV		Personnel^c^		Labs^d^		Other		Total	
Country	Sites (N)	Simple Mean^a^	Weighted Mean	Simple Mean	Weighted Mean	Simple Mean	Weighted Mean	Simple Mean	Weighted Mean	Simple Mean	Weighted Mean
**Rwanda**	30	$114	$125	$67	$51	$15	$15	$37	$25	$232	$216
**Malawi**	30	$66	$72	$29	$23	$5	$8	$36	$34	$136	$137
**Ethiopia**	41	$103	$111	$28	$17	$16	$18	$39	$37	$186	$183
**Zambia**	30	$155	$153	$73	$53	$13	$13	$37	$32	$278	$251
**RSA**	30	$181	$194	$334	$248	$102	$103	$65	$51	$682	$595

a Simple mean calculated across facilities in the sample.

b Weighted mean is weighted by patient-years by facility.

c Direct and indirect personnel are included.

d With the exception of South Africa, lab costs include consumables and reagents only. Lab costs in South Africa are fully-loaded, including both personnel and equipment.

The largest cost components were ARVs (27–56% of total average costs) and personnel (15–49%). Laboratory reagents and consumables averaged 3%–9% of total costs. Other costs, such as nutrition, treatment of OIs, training and equipment and other administrative support services together accounted for 9–27% of total costs.

#### ARV

ARV costs constituted the largest component of average treatment costs across the study sample, representing roughly 50% of average costs in the low and lower middle income countries studied. Costs in South Africa included updated ARV prices, which were renegotiated by the RSA government in early 2010 and are 53% lower than the prices observed during the cost data year.

**Figure 3:**
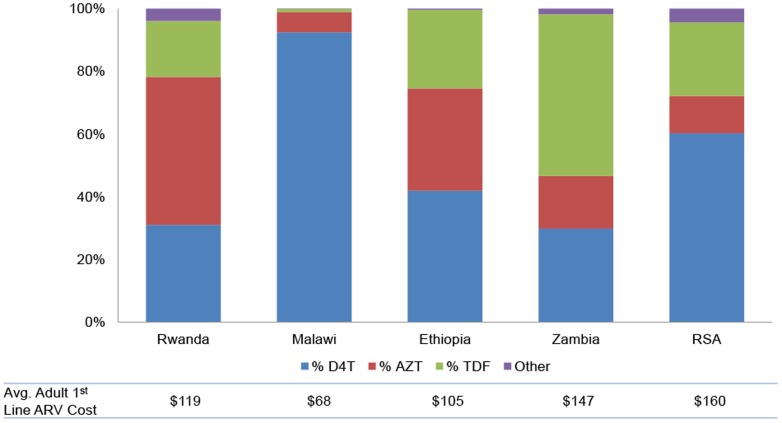
Regimen Selection and Average First Line ARV Costs by Country. Regimen breakdowns are from the cost data year, and do not reflect changes in regimen selection that countries have made since 2009–2011, such as the continued phase-out of d4T.

ARV cost and variability in total cost were related to differences in choice of Nucleoside/Nucleotide Reverse Transcriptase Inhibitors (NRTIs) in the regimen, reflecting the change in regimens that was taking place at the time of the cost data year as a result of shift away from Stavudine (D4T) in line with the 2010 WHO HIV treatment guidelines. Facilities with a higher proportion of patients on Zidovudine (AZT) or Tenofovir (TDF)-based regimens had higher ARV cost per patient-year. Refer to Figure 3 for the regimen breakdown by NRTI across all study countries.

Although second-line (2L) drugs were costly for individual patients, these patients were a small proportion of ART patients in the sampled facilities and associated drug costs made up a small share of average costs. Second-line patients were also concentrated in larger hospitals and centers of excellence, reflecting both more proactive management of treatment failure and referrals from other facilities.

#### Personnel

Variation in direct and indirect personnel (administrative, supervisory and other non-patient facing personnel) cost ppy is correlated to variation in staffing levels, or the number of facility staff allocated to HIV relative to ART patient-years (R^2^ = 0.12, N = 161, p<0.0001). Personnel costs were less correlated to staffing mix, or the ratio of doctors to nurses and clinical officers (R^2^ = 0.029, N = 161, p = 0.031). South Africa had higher personnel costs (mean: $334 ppy), followed by Rwanda and Zambia (mean: $67 and $73 ppy respectively). Ethiopia and Malawi had lower personnel costs (mean: $28 and $29 ppy). Salary levels were higher in South Africa and Zambia, where there is a higher GDP per capita. In Rwanda and Ethiopia, staffing levels were higher. However, Ethiopia had relatively low salary levels. In Malawi, both salary and staffing levels were relatively low. Tables 3 and 4 present salary and staffing data by country.

**Table 3:** Average Annual Salary Levels by Cadre by Country (USD).

Cadre	Rwanda	Malawi	Ethiopia	Zambia	RSA
**Doctor**	$13,732	$12,405	$5,741	$30,106	$90,365
**Clinical Officer**		$4,763	$2,309	$11,255	
**Nurse**	$4,626	$4,376	$1,399	$9,676	$35,907
**CHW**	$336	$1,741	$388	$2,745	$4,032

**Table 4:** Simple Average Staffing Level for HIV per Facility by Cadre by Country (Per 1,000 Patient Years).

Cadre	Rwanda	Malawi	Ethiopia	Zambia	RSA
Doctor	0.8	0.1	0.5	0.3	0.7
Clinical Officer		1.3	0.8	1	
Nurse	7.7	1.7	4.6	2.7	4.5
CHW^a^	5.9	0.2	5.5	0.3	0.7
Other Clinical	2.4	1.7	6.5	5	3.9
**Total Direct Personnel**	**16.8**	**4.9**	**17.9**	**9.3**	**9.9**
**Total Indirect Personnel**	**3.1**	**4.1**	**8.1**	**3.3**	**2.9**
**Total**	**20**	**9**	**26**	**13**	**13**

a CHW refers to community health workers affiliated with facilities.

#### Labs

Excluding South Africa, lab costs were strikingly low, averaging $5–16 ppy for the cost of reagents and consumables. The inclusion of personnel and equipment costs adds only marginally to the lab cost in these countries, increasing them to $10–26 ppy. There were variations in testing frequency, or the number of tests per patient-year, resulting in variation in lab costs. Lab costs in South Africa were significantly higher than all other countries, which can be attributed, at least in part, to a much higher testing frequency of viral load (VL) and other monitoring tests, including kidney and liver function tests. In addition, costs in South Africa include both personnel and equipment, which were included in “personnel” and “other” cost categories in the other four countries.

As illustrated in Table 5, in all five countries, CD4 testing frequency was below standards. Although national guidelines recommend 2 to 3 CD4 tests per patient-year, the average number of CD4 tests received was 1.6 in Rwanda, 0.2 in Malawi, 1.4 in Ethiopia, 1.3 in Zambia, and 1.4 in South Africa. VL was almost non-existent outside of South Africa. Low laboratory network utilization rates resulted in higher costs attributed to each patient. Interviews with facility staff suggested that low testing levels were due in part to shortages of reagent and consumables and frequent machine breakdowns.

**Table 5:** Average Laboratory Monitoring Frequency per ART Patient-Year by Test by Country.

		Rwanda	Malawi	Ethiopia	Zambia	RSA
**CD4**		1.6	0.2	1.4	1.3	1.4
**Viral Load**		0.1	0.1	0	0	1.1
**Fixed Blood Count**		1.2	0.2	1.3	1	1
**Hemoglobin**		0	0.3	0.1	0.1	0.2
**Creatinine**		1.2	0	0.2	0.4	1.3

#### Other

The remaining “other” costs include OI drugs, nutritional support, facility-level training, equipment and supplies (clinical and non-clinical), vehicle and building maintenance and other miscellaneous administrative recurring costs (e.g., transport, security, etc.). These costs vary across countries and facilities, ranging from a simple average of $36 in Malawi (SD: $19) to $65 in South Africa (SD: $32). The average cost of OIs is $6 in Rwanda, $10 in Malawi, $15 in Ethiopia, $11 in Zambia and $15 in South Africa. Non-commodity costs are more variable than OI drug and nutrition costs. This is particularly true in South Africa where administrative, managerial and security costs are $44 ppy on average (SD: $31), compared to OI and nutrition costs which are $20 ppy on average (SD:$9).

### Patient outcomes summary

The results of this unit cost analysis do not represent a normative model of care and must be evaluated together with patient outcomes. As described in Table 6, 14,039 eligible patient-records were collected. The mean attrition rate for established patients during the 12-month cost data year, across all study countries, was 6% (2% in Rwanda, 6% in Malawi, 7% in Ethiopia, 6% in Zambia, and 8% in South Africa). The mean retention rate at 12 months for new patients was 85% (95% in Rwanda, 77% in Malawi, 84% in Ethiopia, 89% in Zambia, and 82% in South Africa). In Zambia, the outcome data proved difficult to assess because the country was in the process of transitioning from paper-based records to a national electronic medical record system during the cost data year, and these results should be interpreted with that in mind. The retention and attrition rates by country are presented in Table 6. Importantly, there is a weak correlation between the cost per patient-year and success at keeping patients alive and retained on treatment at 12 months (R^2^ = 0.005, N = 160, p = 0.004).

**Table 6:** Attrition and Retention Rates by Country.

		New Patients			Established Patients^b^		
Country	Simple Mean Cost	Sites where eligible charts were available	Eligible Patient Charts^a^	Mean retention at 12 months from initiation (95% confidence intervals)	Sites where eligible charts were available	Eligible Patient Charts	Mean attrition over 12 month cost data year (95% confidence intervals)
**Rwanda**	$232	29 of 30	1096	95%±2%	22 of 30	961	2±1%
**Malawi**	$136	30 of 30	1496	77%±3%	23 of 30	1090	6±4%
**Ethiopia**	$186	41 of 41	1944	84%±3%	41 of 41	1862	7±3%
**Zambia**	$278	30 of 30	1568	89%±4%	27 of 30	1299	6±2%
**RSA**	$682	30 of 30	1624	82%±4%	27 of 30	1099	8±4%

a Eligible charts excluded patients that were transferred to another facility.

b Note some sites had not been providing ART for as long and did not have patients that fit the requirements for an established patient.

Patients studied were initiating treatment at relatively low CD4 counts. As illustrated in Table 7, only 27% of patients across the sample, and only 9% of patients in Malawi were found to initiate at a CD4 count over 200 cells/mm^3^. Twenty percent of patients across the sample, and 75% of patients in Malawi, were initiated without a CD4 test. Rwanda was also the only country with a median CD4 count at initiation over 200 cells/mm^3^, initiating 65% of patients at CD4 counts over 200 cells/mm^3^. It is hypothesized that Rwanda's strong pre-ART program contributed to early initiation. Rwanda also had significantly higher retention figures than any other country, with 95% retention of new patients and only 2% annual attrition for established patients.

**Table 7:** Number of Patients in the Sample by CD4 Category (cells/mm^3^).

Nb. of patients by CD4 at Initiation	Rwanda	Malawi	Ethiopia	Zambia	RSA	Total
0–100	148	101	559	437	565	1810
100–200	216	138	666	497	728	2245
200–300	359	102	365	378	190	1394
300–400	336	24	105	163	37	665
No CD4	13	1105	224	64	91	1497
**Mean CD4 (cells/mm^3^)**	231	169	144	158	129	

## Discussion

The $208 simple average facility-level cost ppy for the four low-income and lower-middle income countries in the MATCH study is significantly lower than that seen in previous studies. Previous estimates tended to be from studies conducted in 2004–2008 and ranged from $650 to $1,000 ppy. Menzies et al. conducted a cost analysis at forty three PEPFAR-supported facilities across five countries in 2006–2007, finding a median cost of $880 ppy [5]. A systematic review by Rosen and Long in 2006 identified the average cost of treatment at $850 ppy outside of South Africa [6]. In addition, Marseille et al. found an average cost of $638, of which $428 were facility-level costs, for a sample of forty five PEPFAR-supported Zambian facilities from 2004 to 2008 [7]. A more recent review by Galárraga et al. found median ART unit costs of $792 in low-income countries (LICs) and $932 in lower-middle income countries (LMICs), in studies conducted from 2001–2009 [8]. In 2013, PEPFAR released a study showing that treatment costs at their sites had declined from over $1,100 ppy to about $338 ppy [9].

The MATCH study costs reflect the significant drop in ARV prices that has occurred over the past decade. At the end of 2012, first-line ARV treatment was available in low and lower middle income, generic accessible countries for an average cost of $132 ppy. This represents a significant drop from prices in 2000, when ARVs were $10,000 ppy [10], [11]. South Africa achieved a 53% savings in the 2011 tender alone. Given that ARVs account for half of facility-level costs on average, work to reduce the cost of first- and second-line ARV regimens will continue to be important across LICs and LMICs, particularly as countries have phased out Stavudine in favor of more efficacious but more expensive NRTIs. The Clinton Health Access Initiative and a number of other partners are working on process chemistry and dose optimization efforts aimed at reducing the cost and improving the tolerability of these products.

The results also illustrate other opportunities for improving value for money in HIV spending. Facilities are maintaining established patients alive and on treatment at the relatively low costs. However, this study illustrated variation in retention of new patients, underlining that additional effort should be made to keep new patients in care as countries scale-up ART services. There are potential low-cost interventions to improve quality of care and retention for both new and established patients. For example, reaching 100% coverage of cotrimoxazole would cost roughly $2–10 per patient-year, and could reduce mortality by up to 60% [12].

In South Africa, optimizing facility-level ART service delivery could generate cost savings, given the relatively high cost ppy of $682. In Ethiopia, Malawi, Rwanda and Zambia, given the lower salary and other costs, savings opportunities at the facility-level may be limited in absolute dollar terms. However, there are opportunities for improved efficiency at certain facilities with higher costs. Addressing such inefficiencies is particularly important where limited human resources can be freed for further ART scale-up and non-HIV service delivery.

Overall, facility-level costs likely make up a smaller portion of the 19.1 B in HIV spending than previously understood [13]. Therefore, in order to address broader HIV funding gaps, efforts to improve efficiency may be more productively focused on above facility costs (e.g., management, technical assistance and systems). There may also be a need for better targeting of resources across intervention areas and target populations. These non-treatment programs were not included in the MATCH study.

There are some additional limitations that must be considered in interpreting the results of this study. First, the cost variation between MATCH and previous studies may be due to differences in inclusion criteria. The MATCH study excludes in-patient costs and treatment costs incurred outside the facility, such as community support services, management, technical assistance, systems and overhead costs. While these areas may be significant in some settings, costs and the associated impact on patient outcomes are not well understood.

Second, the “top-down” allocations used for this analysis meant that unit costs were not specific to a given patient, but rather a facility. As a result, expected cost differences between patients at different stages of treatment (e.g., new vs. established) and different CD4 counts or stages of the disease could not be assessed. In addition, allocations informed by interviews were subject to reporting bias. This methodology also does not allow for a detailed examination of quality of care relative to national guidelines. The random sample of facilities included those that did not fully follow standard guidelines (e.g., frequency of CD4 testing) and these costs may be an underestimate for a normative year of treatment. In addition, as described above, the inclusion of normative ARV costs for some facilities, due to data limitations, resulted in some inconsistency across facilities.

Third, data is observational rather than experimentally generated, meaning that relationships between the determinants of cost or retention may not illustrate causation. For example, the impact of community-level adherence support (a determinant of cost and retention) was not captured, but may have had an effect on costs at the facilities studied. Given that the assessment of patient outcomes is based on a retrospective analysis, rather than on data from a prospective study of a treatment cohort, we are also unable to gauge the impact of policy options and quality, such as regimen selection and frequency of laboratory testing over time. Differences in efficiency in program and administration costs were also not captured. While multivariate regression analysis is ongoing, relationships between facility characteristics and cost or outcomes will therefore be limited.

Finally, because the MATCH study sample was randomly drawn from all the ART facilities in each country, facilities did not always have a pre-existing, high-quality information system. As in many similar studies, this results in some expected biases. For example, better-managed facilities may keep good records of personnel training and may falsely appear to spend more on these cost components than facilities where record-keeping is not robust. However, we do not expect that this will have significant impact on the overall findings given the relatively low costs of training and other such areas.

The cost of facility-level treatment will vary across contexts. However, if the costs seen in these five countries are directionally representative of costs in the region, we speculate that the facility-level costs of ART — and therefore the costs of treatment scale-up — are much lower than previously understood. Further analysis is needed on expected changes with scale-up, including up-front investments in systems, as well as the implications of decentralization and scale on costs. Improved understanding of the balance of HIV spending at and above the facility, as well as across HIV programs, is also critical. In an environment of constrained resources, decision makers need this evidence to allocate available funding to underfunded interventions with the greatest potential impact on patient outcomes.
